# Splenic marginal zone lymphoma with monoclonal IgG: A case report

**DOI:** 10.1097/MD.0000000000037158

**Published:** 2024-02-09

**Authors:** Xupai Zhang, Shihui Ren, Nan Zhang, Xiao Wang, Lin Qiu, Haoping Sun, Hai Yi, Fangyi Fan

**Affiliations:** a Department of Hematology, The General Hospital of Western Theater Command, Chengdu, China.

**Keywords:** IgG, lymphoplasmacytic lymphoma/Waldenstrom’s macroglobulinemia (LPL/WM), plasmocytic differentiation, splenic marginal zone lymphoma (SMZL)

## Abstract

**Rationale:**

Splenic marginal zone lymphoma (SMZL), an indolent small B-cell lymphoma, is uncommon, and part of the patients exist plasmocytic differentiation and secrete monoclonal paraproteins including IgM predominantly. SMZL with monoclonal IgG is rarer.

**Patient concerns:**

We report a case of SMZL (49-year-old, male) with monoclonal IgG, MYD88L265P mutation and hepatitis B virus infection.

**Diagnoses:**

The patient was presented to our hospital with aggravating complaints of dizziness, fatigue, postprandial abdominal distension, and night sweats. The diagnosis was confirmed by clinical manifestations, immunophenotype, bone marrow pathology.

**Interventions:**

The patient received rituximab-based chemotherapy and sequential ibrutinib in combination with entecavir.

**Outcomes:**

After 1 year of follow-up, his blood routine examination had returned to normal with normal level of albumin and significantly lower globulin than before, and the spleen was of normal size.

**Lessons:**

We conclude that rituximab-based chemotherapy is the main treatment option for the patients with SMZL, and Bruton’s tyrosine kinase inhibitor has also shown beneficial efficacy.

## 1. Introduction

Splenic marginal zone lymphoma (SMZL) is an indolent small B-cell lymphoma originating from the splenic white pulp, which account for <2% of non-Hodgkin lymphoma.^[1]^ Owing to the paucity of this disease entity, the feature of SMZL has only been characterized partly. The pathogenetic process is chronic and insidious and part of patients with SMZL even have no special clinical symptoms at diagnosis. Despite it originates from the spleen and the splenic hilar lymph nodes are susceptible to involve, peripheral blood and bone marrow are generally involved when confirming the diagnosis of SMZL. Subsequently, peripheral lymph nodes and extra-lymph node organs, liver for instance, can be infiltrated with the disease progressing.^[2]^ Meanwhile, the patients with SMZL frequently combine with autoimmune disease such as autoimmune hemolytic anemia, Sjogren syndrome and immune thrombocytopenia.^[3]^ In spite of its indolence, approximately 10% of individuals with SMZL probably convert into aggressive diffuse large B-cell lymphoma.^[4]^ Furthermore, about one third of patients exist plasmocytic differentiation and secrete monoclonal paraproteins including IgM predominantly. Nevertheless, very few reports about the cases of SMZL with plasmocytic differentiation, especially accompanying with monoclonal IgG, are available at present. We currently report a case of SMZL with monoclonal IgG and meanwhile conduct literature review in order to improve the understanding of the diagnosis, differential diagnosis and treatment of this uncommon entity.

## 2. Case presentation

**Figure 1.**
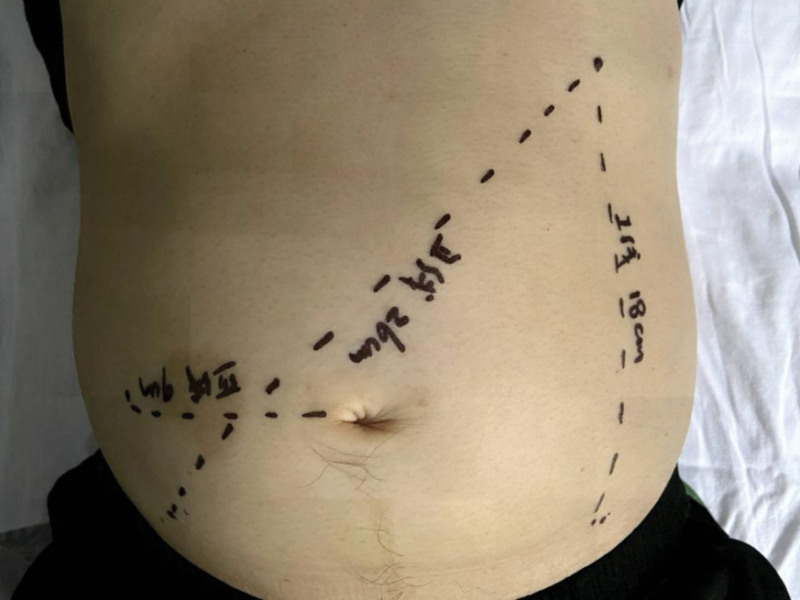
Physical examination of the spleen. The prominent splenomegaly measured 18 cm, 26 cm and +9 cm in line I to III, respectively.

**Figure 2.**
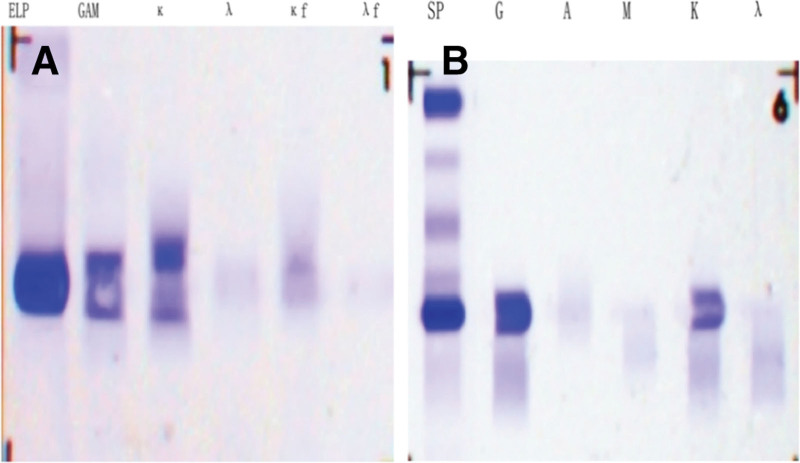
The electrophoresis of urine Bence-Jones protein and the serum immunofixation electrophoresis. (A) The urine Bence-Jones protein was positive for kappa light chain. (B) The serum immunofixation electrophoresis suggested the monoclonal immunoglobulin was IgG-kappa type.

A 49-year-old male was presented to our hospital with aggravating complaints of dizziness, fatigue, postprandial abdominal distension and night sweats 3 days before admission in September 2021. The patient had no fever, weight loss or abdominal pain and denied past medical history such as hepatitis B virus (HBV), hepatitis C virus (HCV), and human immunodeficiency virus infection. He also had no surgical history or family history of analogous hematological disorders. Upon admission, physical examination revealed the patient had pale appearance and bilateral axillary and inguinal lymphadenectasis. The liver was not beyond the rim of his ribs. However, we discovered that he suffered from prominent splenomegaly (18 cm, 26 cm and +9 cm in line I to III, respectively) (Fig. 1). Blood routine test showed mildly decreased leukocyte (3.30 × 10^9^/L), significantly reduced hemoglobin (56 g/L), and normal platelet (115.00 × 10^9^/L). The ratio of neutrophil was 21.30% (normal range: 40.00–75.00%), while the ratio of lymphocyte was 63.00% (normal range: 20.00–50.00%). The blood biochemical detection showed low albumin (21.0 g/L), high globulin (87.3 g/L), normal lactate dehydrogenase (106 U/L), and elevated β2 microglobulin (5.43 μg/mL). Meanwhile, quantitative detection of immunoglobulin displayed extremely elevated IgG (55.08 g/L, normal range: 7.00–16.00 g/L), normal IgM (0.98 g/L, normal range: 0.40–2.30 g/L), and slightly reduced IgA (0.39 g/L, normal range: 0.70–4.00 g/L). His urine routine examination showed the urinary protein was positive (++), and Bence-Jones protein was also positive for kappa light chain (Fig. 2A). Especially, serum protein electrophoresis revealed M-protein bands, and serum immunofixation electrophoresis suggested that the monoclonal immunoglobulin was IgG-kappa type (Fig. 2B). The direct and indirect antiglobulin test and autoimmune antibody test were negative. In addition, the patient was measured for HBV surface antigen (+), HCV antibody (−), and human immunodeficiency virus (−), and the deoxyribonucleic acid quantitation of HBV failed to be detected (below 1.00 × 10^3^ IU/mL). Further abdominal ultrasonography (US) detected evident splenomegaly with an intercostal thickness of roughly 9.5 cm, and the US of superficial lymph nodes showed bilateral axilla and groin existed lymphadenectasis (the maximum of axilla: 15 × 7 mm on the left, 28 × 6 mm on the right, the maximum of groin: 21 × 4 mm on the left, 16 × 3 mm on the right, respectively). For some reasons, the patient refused to finish the positron emission tomography/computed tomography.

**Figure 3.**
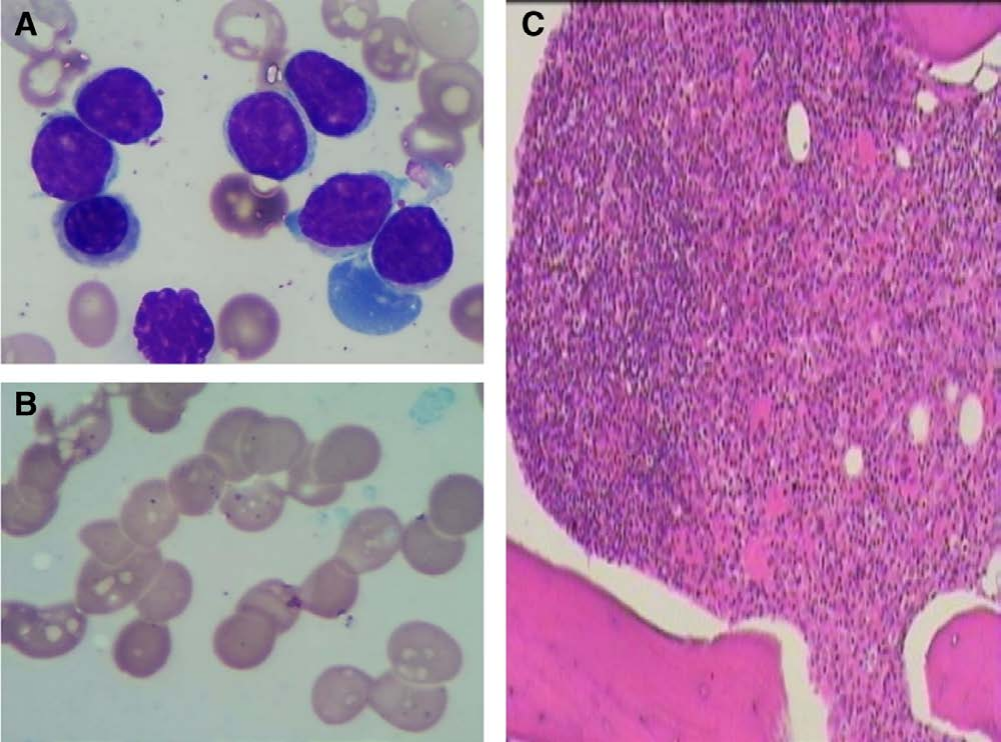
The bone marrow morphology and bone marrow biopsy of the patient. (A) The bone marrow smear showed the lymphocytes (mostly mature lymphocytes) abnormally proliferated. (B) The bone marrow smear revealed the mature-phase erythrocytes were in rouleau formation. (C) The bone marrow biopsy revealed extraordinarily active proliferation with high proportion of mature lymphocytes and dispersed plasma cells.

**Figure 4.**
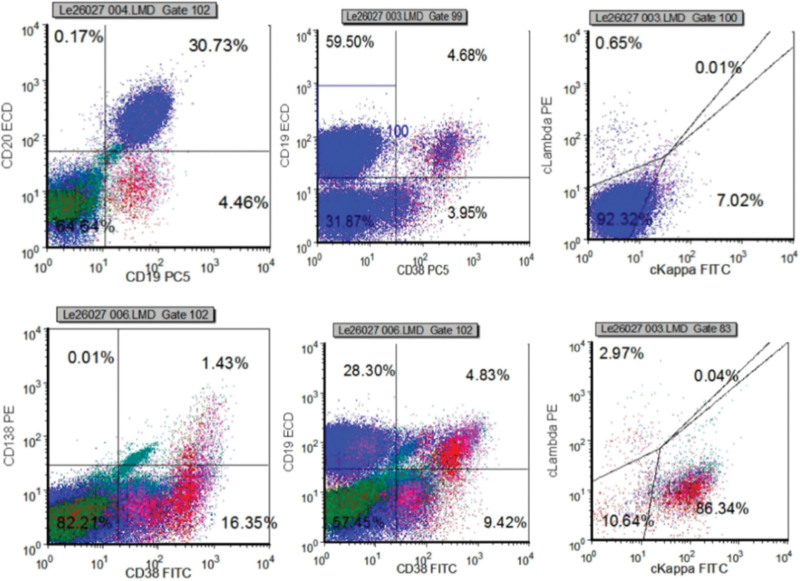
The part results of the flow cytometry (FCM) analysis. The FCM analysis of bone marrow displayed that about 30.73% of abnormal B cells were positive for CD45, CD19, CD20, CD22, CD79b (part), CD13 (part), HLA-DR and negative for CD5, CD10, FMC7, CD23, CD200, CD103, CD38, CD138, CD56, CD2, CD3, CD4, CD34, CD33, CD11b; about 3.6% of monoclonal plasma cells expressed CD38, CD138, CD19 and did not express CD56, CD20, the cytoplasmic immunoglobulin kappa light chain of which was restrictively expressed.

Additionally, a bone marrow morphology revealed that all nucleate cells (ANC) proliferation was obviously active, among which lymphocytes (mostly mature lymphocytes, occasionally see smear cells) abnormally proliferated and accounted for 39.5% of ANC; granulocytes proliferation, particularly made up 37.5% of ANC, was modestly reduced and primarily consisted of mature-phase cells with normal morphology; erythrocytes proliferation, accounted for 20% of ANC, was active and the mature-phase erythrocytes could be partly saw in rouleau formation; plasma cells were present in 1% of ANC (Fig. 3A and B). A bone marrow biopsy revealed extraordinarily active bone marrow proliferation with high proportion of mature lymphocytes and dispersed plasma cells; the reticulin stain failed to display collagen fibrosis in the interstitial bone marrow (Fig. 3C). Noteworthy, the immunohistochemical (IHC) result discovered 2 categories: one with lymphocytes, visualized as intrasinusoidal infiltration, CD20 (+), CD5 (−), Cyclin D1 (−), CD103 (−), CD23 (−), SOX-11 (−); the other with plasma cells, showing diffuse hyperplasia, CD38 (+), CD138 (+), kappa (+), lambda (−). Then, the flow cytometry (FCM) analysis of bone marrow revealed that about 30.73% of abnormal B cells were positive for CD45, CD19, CD20, CD22, CD79b (part) and negative for CD5, CD10, FMC7, CD23, CD200, CD103; about 3.6% of monoclonal plasma cells expressed CD38, CD138, CD19, the cytoplasmic immunoglobulin kappa light chain of which was restrictively expressed (Fig. 4). Meanwhile, the next generation sequencing of bone marrow showed positive for MYD88^L265P^ (the variation frequency: 12.3%) and negative for CXCR4, BRAF, and NOTCH2. In addition, the core needle biopsy of lymph node in the right axilla conformed to indolent small B-cell lymphoma with lymphocytes CD20 (+), CD5 (+, minority), CD10 (−), Cyclin D1 (−), Bcl-2 (+), Ki-67 (+, 5%), plasma cells CD138 (+), kappa (+), lambda (−) and follicular dendritic cells (FDC) CD23 (+).

According to medical history, clinical manifestation, and related inspection of the patient combined with the minimum diagnostic criteria of SMZL by the Splenic B-cell Lymphoma Group (SBLG), a diagnosis of SMZL was ultimately ascertained.^[5]^ Subsequently, he received 4 cycles of chemotherapy with rituximab (600 mg/day, day + 1), cyclophosphamide (1200 mg/day, day + 1), vindesine (4 mg/day, day + 1), and prednisone (60 mg/day, day +1 to +5) (R-CVP) accompanying with entecavir (0.5 mg/day). Then the patient was given sequential ibrutinib (560 mg/day) and did experience related side effects to reduce the dose. After treatment, the symptoms of fatigue and postprandial abdominal distension diminished with the blood routine examination close to normal and the spleen significantly smaller than before. After 1 year of follow-up, his blood routine examination had returned to normal with normal level of albumin and significantly lower globulin than before, and the spleen was of normal size by the US. Furthermore, this patient is currently being constantly monitored.

## 3. Discussion

SMZL is classified as a subset of independent mature B-cell derived lymphoma in the 2016 World Health Organization classification of the lymphoid neoplasms. Based on the guideline of SMZL from SBLG, the minimum diagnostic criteria of SMZL should meet 1 of the following 2: (i) the histological characteristics of the spleen specimen and the chronic lymphocytic leukemia immunophenotype score of no more than 2 points; (ii) typical peripheral blood and bone marrow morphology, immunophenotype and intrasinusoidal infiltration of CD20 + cells in bone marrow tissue if the spleen specimen is unavailable.^[5]^ Generally, the peripheral blood or bone marrow smear of the patient with SMZL may be found distinctive polar villous lymphocytes, but the morphological characteristic can be missed due to the anticoagulant-induced loss of villi on the surface of tumor cells and the inexperience of some examiners. Moreover, some data revealed that the incidence of intrasinusoidal infiltration of CD20 + cells in bone marrow tissue of the patients with SMZL was approximately 30%.^[6]^ The intrasinusoidal infiltration, serving as a characteristic pathological feature, occurred predominantly in the early stage of the disease and developed into nodular interstitial infiltration in the later stages of the disease. Some studies reported that 6% to 21% of SMZL patients had been described to exist the MYD88^L265P^ mutation, which was thought to increase the occurrence of plasmocytic differentiation.^[7]^ Owing to lack of the spleen specimen, the patient met the second minimum diagnostic criteria for SMZL including an increased proportion of lymphocytes in peripheral blood and bone marrow, a 1-point chronic lymphocytic leukemia score, and characteristic CD20 + cells with intrasinusoidal infiltration. Meanwhile, both bone marrow FCM analysis and biopsy suggested the presence of abnormal B lymphocytes and monoclonal plasma cells, and the serum immunofixation electrophoresis suggested IgG-kappa type monoclonal immunoglobulin with MYD88^L265P^ mutation. Hence, a diagnosis of SMZL with monoclonal IgG could be confirmed.

During the diagnostic process, SMZL, a subset of B-cell chronic lymphoproliferative disorders, needs to be differentiated from other B-cell chronic lymphoproliferative disorders such as chronic lymphocytic leukemia/small cell lymphoma, mantle cell lymphoma, follicular lymphoma, and lymphoplasmacytic lymphoma/Waldenstrom’s macroglobulinemia (LPL/WM). Although they all may accompany with plasmocytic differentiation, we can differentiate them by means of morphology, immunophenotype, and molecular biology. The negative CD5, CD23, and CD200 may exclude the diagnosis of chronic lymphocytic leukemia/small cell lymphoma, and the negative CD5, Cyclin D1, FMC7, and SOX-11 can exclude the possibility of mantle cell lymphoma. The immunophenotype of the patient with follicular lymphoma is usually positive for CD10 and Bcl-6 (IHC).

However, SMZL with plasmocytic differentiation, especially in patients with MYD88^L265P^ mutation, is particularly indistinguishable from LPL/WM due to lack of splenic pathological results in most patients. Differences between SMZL and LPL/WM include, but are not limited to, clinical features, morphology, immunophenotype, molecular biology, cytogenetics, and pathology. The patient with SMZL commonly presents with marked splenomegaly, higher number of leukocytes, and lack of hyper viscosity symptoms, while the patient with LPL/WM is susceptible to hyper viscosity symptoms and autoimmune diseases. Morphologically, the bone marrow of SMZL appears increased proportion of lymphocytes mostly with abundant cytoplasm, deep chromatin, and inconspicuous nuclei, as well as villous lymphocytes, while LPL/WM is consisted of small lymphocytes, plasmacytoid lymphocytes, and plasma cells, and additional mastocytes are visualized without villous lymphocytes. Although, SMZL with plasmocytic differentiation and LPL/WM both have abnormal lymphocytes and monoclonal plasmacytes in the FCM analysis of bone marrow, research had shown that the level of CD138 expression in the bone marrow of patients with LPL/WM was dramatically higher than that of patients with SMZL.^[8]^ In addition, the significantly higher expression level of CD180 in SMZL patients compared to LPL/WM was expected to be a specific positive marker for SMZL.^[9]^ In molecular biology, MYD88^L265P^ mutation was in 6% to 21% of patients with SMZL and in over 90% of LPL/WM patients. Moreover, NOTCH2 (10–25%) and KLF2 (12–44%) mutations were highly prevalent molecular lesions in SMZL,^[10]^ while about 40% of LPL/WM patients had CXCR4 mutations. Several studies had shown that the expression levels of EME2 and USP24 were significantly higher in SMZL patients than in other B-cell lymphomas.^[11]^ Cytogenetically, 7q deletion, trisomy 3, and 12q amplification were found in patients with SMZL,^[12]^ while 6q21 deletion was observed in LPL/WM.^[13]^ Pathologically, the bone marrow tissue of the patients with SMZL appeared intrasinusoidal infiltration of CD20 + cells, while the invasion of the bone marrow commonly manifested as paratrabecula in LPL/WM. Reactive mastocytes hyperplasia in bone marrow biopsies was characteristically found in LPL/WM but not in SMZL. In contrast to the patients with LPL/WM who predominantly had monoclonal IgM, those with SMZL with plasmocytic differentiation had a much smaller percentage of IgG, IgA, and nonsecretory immunoglobulins. Besides, B lymphocytes undergo somatic mutations and sequence rearrangements in the VDJ region of the immunoglobulin heavy chain region (IGHV) during development, resulting in the formation of some different B cell receptors (BCR).^[14]^ For SMZL patients, IGHV1-2 rearrangements in the V region were specific, mostly without antigen selection, and have relatively longer CDR3 sequences, whereas LPL/WM patients were often observed IGHV2-23 rearrangements, mostly with antigen selection, and have shorter CDR3 sequences.^[15]^ Furthermore, the high-throughput technology like Nano string Counter may suggest the expression profile characteristics of specific lymphoma subtypes by detecting the signature genes of different lymphomas in combination with pre-models, thus playing a guiding role in the differentiation of SMZL and LPL/WM.

Currently, patients with SMZL with no significant clinical symptoms, noticeable lymphadenectasis, hepatosplenomegaly, or progressive hemocytopenia can be watched and waited. For HCV (+) patients with splenomegaly, antiviral therapy has shown better efficacy and makes it achieve complete remission. The rituximab or combined chemotherapy has demonstrated efficacy in HCV (−) patients with symptomatic splenomegaly, and rituximab shows amazing therapeutic effect not only in induction therapy but also in maintenance therapy.^[16]^ In contrast, splenectomy is mainly indicated for patients with splenomegaly, significant hemocytopenia, ineffective rituximab, and bearable surgery. Small molecular Bruton’s tyrosine kinase inhibitor, such as ibrutinib, reduces the malignant proliferation of B lymphocytes and induce apoptosis by inhibiting the activation of BCR signaling pathway. Some studies had shown that ibrutinib monotherapy was effective in treating relapsed and refractory marginal zone lymphoma, particularly in SMZL patients. Compared with other non-Hodgkin lymphomas, the incidence rate of HBV infection was obviously higher in SMZL patients, and the positive HBV surface antigen was a risk factor scoring system to determine the prognosis of SMZL patients.^[17]^ The patient with SMZL could achieve complete remission with anti-HBV therapy, indicating that HBV infection was associated with the development of SMZL. In our present case, the patient suffered from fatigue and postprandial abdominal distension with splenomegaly and severe anemia and had therapeutic indications. After rituximab-based chemotherapy and sequential ibrutinib in combination with entecavir, the blood routine test had returned to normal with normal level of albumin, significantly lower globulin, and normal sized spleen.

In conclusion, the cases of SMZL with plasmocytic differentiation are uncommon, which are usually diagnosed by clinical manifestations, immunophenotype, bone marrow pathology if no spleen specimen can be available. SMZL with plasmocytic differentiation, especially in patients with MYD88^L265P^ mutation, is particularly indistinguishable from LPL/WM. Importantly, rituximab-based chemotherapy is the main treatment option, and the Bruton’s tyrosine kinase inhibitor has also shown beneficial efficacy.

## Acknowledgments

We appreciate the financial support by the Basic and Frontier Research Project of General Hospital of Western Theater Command (Grant No. 2021-XZYG-C46).

## Author contributions

**Conceptualization:** Xupai Zhang, Haoping Sun, Hai Yi, Fang-Yi Fan.

**Data curation:** Xupai Zhang.

**Formal analysis:** Xupai Zhang.

**Writing – original draft:** Xupai Zhang.

**Writing – review & editing:** Xupai Zhang, Shihui Ren, Nan Zhang, Xiao Wang, Lin Qiu, Haoping Sun, Hai Yi, Fang-Yi Fan.
